# Ultrasound-assisted extraction of protein from Bombay locusts and its impact on functional and antioxidative properties

**DOI:** 10.1038/s41598-021-96694-w

**Published:** 2021-08-27

**Authors:** Passakorn Kingwascharapong, Manat Chaijan, Supatra Karnjanapratum

**Affiliations:** 1 Department of International Professional in Culinary Art, Faculty of International Hospitality Industry, Dusit Thani College, Bangkok, 10250 Thailand; 2 Food Technology and Innovation Research Centre of Excellence, Department of Food Industry, School of Agricultural Technology and Food Industry, Walailak University, Thasala, Nakhon Si Thammarat, 80161 Thailand; 3 School of Food Industry, King Mongkut’s Institute of Technology Ladkrabang, Ladkrabang, Bangkok, 10520 Thailand

**Keywords:** Biochemistry, Biophysics, Chemistry

## Abstract

Impact of ultrasound-assisted process (UAP) on yield, functional properties, antioxidant properties and molecular characteristics of protein extracted from Bombay locusts (BL) (*Patanga succinta* L.) was studied. Different conditions of UAP were implemented for different amplitudes (40–60%) and times (10–30 min) during aqueous extraction. Notably, UAP could enhance yield and protein recovery, compared with those from typical process (TP) (continuously stirred at 100 rpm at room temperature for 1 h). UAP conditions used governed the change of surface hydrophobicity and free α-amino content of BL. UAP could improve solubility of BL, especially at pH levels higher than 2. UAP had no significant (*p* > 0.05) detrimental effects on foaming capacity and stability of BL. Nevertheless, UAP, particularly at 50–60% amplitudes, affected the emulsion activity and stability of BL. UAP provided BL with high radical scavenging activities and good electron donating ability, especially that from 60% amplitude for 20 min (UAP-60/20). UAP-60/20 showed the impact on change of isoelectric point and molecular characteristic monitored by Fourier transform infrared (FTIR) of BL, compared to those from TP. In addition, BL was also an excellent source of both essential and nonessential amino acids. Therefore, UAP potentially enhanced BL extraction efficiency, resulting the BL with good functional and antioxidative properties.

## Introduction

Recently, alternative protein sources have become a leading concern in food product development, stimulating enthusiasm for plant-based and insect-based proteins^1^. Proteins extracted from insects are becoming one of the most important alternative protein sources and have emerged as a solution of alternative protein production, since edible insects contain high levels of nutrients especially protein (15–82% dry basis) and fat (4–77% dry basis)^2,3^. However, the insect species, the development stage of the insect, season and the insect feed could vary their nutrient content. Their nutrient content of insect protein extract could also vary with the extraction processes used such as extraction techniques applied, process flow and condition conducted as well as powdering and drying methods used^2,4,5^. Ultrasonication is considered as a green technology with being an improvement on traditional extraction processes^6^, which has been gaining much attention. The main factor of ultrasonication that governs protein extraction efficiency is the cavitation effects of ultrasound, which could cause pressure fluctuation leading to explosion of microscopic bubbles. This phenomenon leads to disruption of tissue and decreases the particle size, thus providing intensification of mass transfer^7–9^.

It has been reported that ultrasound-assisted processing (UAP) increased protein extraction from various sources including the skins of clown featherback fish (*Chitala ornate*)^9^, rice bran^10^, and edible insects^11^. Also, UAP has been shown to change the molecular characteristics of proteins in a way that can improve their functional and antioxidative properties including their solubility, foaming and emulsion properties^8,12^, thermal properties^9^ and antioxidative activity^13^. However, the application of ultrasound under inappropriate conditions could also have detrimental effects on protein characteristics that consequently could affect their quality^7,13^.

Bombay locust (BL) (*Patanga succincta* L.) is a major agricultural pest in South and Southeast Asia, including Thailand, where it is also popular as human food^14^. Lombardi et al.^15^ reported that BL was a valuable source of high-quality proteins, minerals, vitamins and fiber. Indriani, et al.^16^ found that hexane defatting could effectively improve protein content of BL powder up to 78.7% and provided the excellent applicability to bakery products. Chatsuwan et al.^17^ successfully extracted the protein from BL by using water, in which the resulting protein had a good antioxidative activity and functional properties. These could indicate the benefit of BL protein for human nutrition as a food ingredient. Nevertheless, the traditional extraction techniques, which they used, gave low yields (7.35% wet basis) and the extraction process was time-consuming (12 h), making the process inappropriate for application in the food industry.

The aim of this study was, therefore, to investigate possible improvement of the efficiency of protein extraction from BL using water extraction with UAP as a green process (without chemical added), by varying its amplitude and processing times in order to optimize yield and the characteristics of the extracted proteins. In addition, functional properties, antioxidative activity and molecular characteristics of the extracted proteins were evaluated.

## Materials and methods

### Chemicals

Bathophenanthrolinedisulphonic acid, 2,2′-azinobis (3-thylbenzothiazoline-6-sulfonic acid) (ABTS), 6-hydroxy-2,5,7,8-tetramethyl-chroman-2-carboxylic acid (Trolox), 2,4,6-trinitrobenzenesulphonic acid (TNBS), and 3-(2-pyridyl)-5,6-diphenyl-1,2,4-triazine-4′,4′′-disulfonic acid sodium salt (ferrozine) were purchased from Sigma-Aldrich Chemical Co. (St. Louis, MO, USA). Bovine serum albumin (BSA) was obtained from Sigma Aldrich Chemical Co. (St. Louis, MO, USA) and hexane was from Macron Fine Chemicals TM (Dublin, Ireland). All chemicals used in this study were of analytical grade.

### Preparation of Bombay locust powder

Frozen Bombay locusts with less than 3-month storage time were obtained from a commercial supplier (Mr. BUC FOOD, Phra Nakhon Si Ayutthaya, Thailand). Samples were cleaned, dried and powdered as described by Indriani et al.^16^ with slight modification. Briefly, sample powder was mixed with hexane at a ratio of 1:5 (w/v) and continuously stirred for 3 h, with the solvent changed every hour. The mixture was then centrifuged in a refrigerated centrifuge (Allegra®x-12r, Beckman Coulter, Inc., USA) at 3000 rpm for 20 min at 25 °C. The sediment was collected and dried on an aluminum foil tray in a fume hood overnight. The resulting Bombay locust powder was used for protein extraction.

### Extraction of Bombay locust proteins

The water extraction without chemical addition was used for protein extraction as a green process. Two processes of protein extraction from BL were compared. The first used water extraction and is called the “typical process” and is similar to that used in commercial extraction of protein. The second used water extraction with ultrasound-assisted process and is called the ultrasound-assisted process.

#### Typical process

BL proteins were extracted using a typical method (TP) as described by Chatsuwan, et al.^17^ with slight modification. The insect powder (20 g) was mixed with distilled water at a ratio of 1:20 (w/v) in a 600-mL beaker. The suspension was then continuously stirred using an overhead stirrer (model W20.n, IKA®-Werke GmbH & CO.KG, Stanfen, Germany) at 100 rpm at room temperature for 1 h. The suspension was centrifuged at 5000 rpm for 15 min at 4 °C using a high-speed refrigerated centrifuge (model CR22N Hitachi, Tokyo, Japan). The undissolved debris layer was removed, and the supernatant was collected and freeze-dried in a freeze-dryer (CoolSafe 55-4 Pro Freeze dryer, ScanLaf A/S, Lynge, Denmark) and the resulting protein was referred to as Bombay locust protein extracted by typical process.

#### Ultrasound-assisted process (UAP)

The insect powder (20 g) was mixed with distilled water at a ratio of 1:20 (w/v) in a 600-mL beaker and then subjected to ultrasonication using an ultrasound reactor model Vibra-Cell (Sonics & Material, Inc, Newtown, CT, USA) and a flat tip probe (25 mm diameter) with a maximum amplitude of 70 micron at 100% amplitude. The reaction was carried out at a power of 750 W, a single frequency of 20 kHz in a pulse mode of alternating 5 s on-time and 5 s off-time. The extraction was conducted with ultrasound at 40, 60 or 80% under different ultrasonication times (10, 20 or 30 min). During ultrasonication, the temperature of mixture was controlled at 25–35 °C monitored by IKA® ETS-D5 temperature controller (IKA-Werke GmbH & Co., German), using an ice bath. After ultrasonication treatment, the extraction was carried out with continuous stirring using an RW 20.n overhead stirrer (IKA®-Werke GmbH & Co. KG, Staufen, Germany) until the end of extraction process (1 h). Each mixture was then centrifuged, and the supernatant collected and freeze-dried as indicated above.

### Analyses

#### Yield of solids

The solids yield was calculated as percentage of the weight of extracted Bombay locust protein in comparison with the weight of insect powder, as follows:

$$\mathrm{Yield\,of\,Solids }(\mathrm{\%})=\frac{\mathrm{Weight\,of\,dried\,extracted\,protein }(\mathrm{g})}{\mathrm{Weight\,of\,insect\,powder }(\mathrm{g})}\times 100$$

#### Protein recovery

Protein recovery was calculated as a percentage of the total extracted protein in comparison with that of the initial insect powder, and was determined using the Kjeldahl method^18^ with the conversion factor 6.25.

$$\mathrm{Protein\,recovery }(\mathrm{\%})=\frac{\mathrm{Protein\,content\,of\,extracted\,insect\,protein }(\mathrm{g})}{\mathrm{Protein\,content\,of\,insect\,powder}(\mathrm{g})}\times 100$$

#### Surface hydrophobicity

Surface hydrophobicity (SHP) of BL samples was determined using a 8-anilino-1-naphththalene sulfonic acid (ANS) as a probe^19^. Relative fluorescence intensity (RFI) of both the buffer (blank) and each protein solution (0.05–0.50 mg/mL) was measured using a fluorescence spectrophotometer (F-2700 Hitachi, Japan) at 390 nm (excitation wavelength) and 480 nm (emission wavelength). The initial slope of the plot of standardized net RFI values versus percentage protein concentration was expressed as the surface hydrophobicity.

#### Free α-amino group content

The free α-amino group content was determined according to the method of Benjakul and Morrissey^20^. Briefly, the sample solution (125 μL) was mixed thoroughly with 2.0 mL of 0.2125 M phosphate buffer (pH 8.2), followed by the addition of 1.0 mL of 0.01% TNBS solution. The mixture was then incubated at 50 °C for 30 min in the dark. The reaction was terminated by adding 2.0 mL of 0.1 M sodium sulphite and cooled down at room temperature for 15 min. The absorbance was measured at 420 nm using a spectrophotometer (model UV-1800, Shimadzu, Kyoto, Japan) and the α-amino group content was expressed in terms of L-leucine.

### Evaluation of functional properties of Bombay locust proteins

#### Protein solubility

Protein solubility of protein solutions (10 mg/mL) was determined at different pH levels (2, 4, 6, 8, and 10) that were adjusted by using 1 M HCl or 1 M NaOH. After being centrifuged at 5,000 rpm for 10 min, the protein content in the supernatant was measured according to the method described by Lowry et al.^21^ using bovine serum albumin as a standard. The relative solubility was calculated in comparison to the protein content of supernatant from those samples solubilized in 0.5 M NaOH (100% relative solubility).

#### Foaming properties

Foaming capacity (FC) and foam stability (FS) of the protein samples were determined using the method described by Chatsuwan et al.^17^. FS was evaluated for the foam samples after leaving at room temperature for 60 min. FC and FS were then calculated using the following equations:

$$\mathrm{FC}(\mathrm{\%})=\frac{{V}_{T}}{{\mathrm{V}}_{0}}\times 100$$

$$\mathrm{FS}(\mathrm{\%})=\frac{{V}_{60}}{{\mathrm{V}}_{0}}\times 100$$

where V_T_ is the total volume after whipping, V_0_ is the original volume before whipping, V_60_ is the total volume after leaving at room temperature for 60 min.

#### Emulsifying properties

Emulsion activity index (EAI) and emulsion stability index (ESI) were determined according to the method described by Chatsuwan et al.^17^. Emulsion sample was prepared by homogenizing (Model T25 basic; IKA) soybean oil (2 mL) and protein solution (5 mg/mL, 6 mL) at 20,000 rpm for 1 min. ESI was evaluated for the emulsion after leaving at room temperature for 10 min. Absorbance at 500 nm (A_500_) of the resulting emulsion under appropriate dilution (using 0.1% (w/v) Sodium dodecyl sulfate) was measured using a spectrophotometer (UV-1800, Shimadzu, Japan). EAI and ESI were calculated as follows:

$$\mathrm{EAI}({m}^{2}/\mathrm{g})=\frac{2\times 2.303\times \mathrm{A}\times \mathrm{DF}}{\mathrm{l\o C}}$$

where A is A_500_, DF is dilution factor, l is path length of cuvette (m), ø is oil volume fraction, C is protein concentration in the aqueous phase (g/m^3^),

$$\mathrm{ESI}(\mathrm{min})={A}_{0}\times (\frac{t}{\Delta A})$$

where ΔA is A_0_ − A_10_, t is 10 min.

### Evaluation of antioxidative activities of Bombay locust proteins

#### DPPH radical scavenging activity

DPPH radical scavenging activity was determined by DPPH assay as described by Binsan, et al.^22^ with a slight modification. Sample (1.5 ml) was added with 1.5 ml of 0.15 mM 2,2-diphenyl-1-picryl hydrazyl (DPPH) in 95% ethanol. The mixture was mixed vigorously and allowed to stand at room temperature in the dark for 30 min. The absorbance of the resulting solution was measured at 517 nm using a UV-1601 spectrophotometer (Shimadzu, Kyoto, Japan). A standard curve was prepared using Trolox in the range of 10–60 μM. The activity was expressed as μmol Trolox equivalents (TE)/g of the sample.

#### ABTS radical scavenging activity

ABTS radical scavenging activity was determined by ABTS assay as per the method of Binsan et al.^22^ with a slight modification. Fresh ABTS solution (7.4 mM ABTS solution in 2.6 mM potassium persulphate solution) was prepared for each assay by mixing 1 mL ABTS solution with 50 mL distilled water in order obtain an absorbance of 1.1 ± 0.02 units at 734 nm using a UV-1601 spectrophotometer (Shimadzu, Kyoto, Japan). Sample (150 μL) was mixed with 2850 μL of ABTS solution and the mixture was left at room temperature for 2 h in dark. The absorbance was then measured at 734 nm using the spectrophotometer. A standard curve of Trolox ranging from 50 to 600 μM was prepared. The activity was expressed as μmol Trolox equivalents (TE)/ g of the sample.

#### FRAP (Ferric reducing antioxidant power)

FRAP was assayed according to Benzie and Strain (1996). Stock solutions included 300 mM acetate buffer (pH 3.6), 10 mM TPTZ (2,4,6-tripyridyl-s-triazine) solution in 40 mM HCl, and 20 mM FeCl_3_ · 6H_2_O solution. A working solution was prepared freshly by mixing 25 mL of acetate buffer, 2.5 mL of TPTZ solution and 2.5 mL of FeCl_3_ · 6H_2_O solution. The mixed solution was incubated at 37 °C for 30 min and was referred to as FRAP solution. A sample (150 μL) was mixed with 2850 μL of FRAP solution and kept for 30 min in dark. The ferrous tripyridyltriazine complex (coloured product) was measured by reading the absorbance at 593 nm. The standard curve was prepared using Trolox ranging from 50 to 600 μM. The activity was expressed as μmol Trolox equivalents (TE)/ g of the sample.

### Characterization of Bombay locust proteins extracted by selected UAP condition

The BL extracted by UAP at 60% amplitude for 20 min (UAP-60/20) showed a high yield with good functional and antioxidative properties, and was selected for future study. BL from UAP-60/20 was then characterized in comparison with BL from typical process (TP).

#### Zeta potential

Zeta potential of the protein samples at different pH levels (2–12) was evaluated using a zeta potential analyzer model ZetaPALs (Brookhaven Instruments Co., NY, USA), which was connected to an auto-titrator model BI-ZTU (Brookhaven Instruments Co., Holtsville, NY, USA). pH showing zero potential was determined as isoelectric point of sample tested.

#### Fourier transform infrared (FTIR) spectroscopy

FTIR analysis was conducted according to the method described by Karnjanapratum, et al.^23^ with modifications. Dehydrated collagens were subjected to ATR-FTIR model Equinox 55 (Bruker, Ettlingen, Germany). Spectra ranging from 650 to 4000 cm^−1^ with a resolution of 4 cm^−1^ were measured. Analysis of the spectra was carried out using OPUS 3.0 software (Bruker, Ettlingen, Germany).

#### Amino acid composition

Each sample was hydrolyzed using 0.4 M methyl sulfonic acid under reduced pressure at 100 °C for 22 h to prevent tryptophan decomposition. The hydrolysates were neutralized with 3.5 M NaOH and diluted with 0.2 M citrate buffer (pH 2.2). A 10 μL aliquot was placed in an amino acid analyzer (JLC-500/V AminoTacTM, JEOL Inc., USA). The amino acid composition was expressed as g/100 g sample^23^.

### Statistical analysis

Experiments were carried out in triplicate and the resulting data were subjected to one-way analysis of variance (ANOVA). Comparison of means was analyzed using Duncan’s Multiple Range Tests^24^. Statistical analysis was performed using the Statistical Package for Social Science (IBM SPSS Statistics, IBM, New York, USA).

## Results and discussions

### Yield of solids and protein recovery

Yield of solids and protein recovery are important parameters that can be used in identifying the efficacy of extraction processes^7^. It was found that UAP significantly increased yield of BL solids (*p* ≤ 0.05) at all the conditions tested (32.48–38.62%), compared with that from TP (22.27%) (Table 1). Solids yield of UAP significantly increased (*p* ≤ 0.05) with increasing of ultrasonication time and amplitude used. However, ultrasonication with a longer period did not significantly (*p* > 0.05) enhance the solid yield, compared at the same amplitude, especially at 60% amplitude. It was noted that UAP at 60% amplitude gave significantly (*p* ≤ 0.05) the highest yield of solids for every ultrasonication time tested. This is consistent with the results reported by Jain and Anal^25^ who showed that cavitation from ultrasonic assisted extraction could facilitate the disruption and degradation of insect powder matrices. This is because ultrasonication favors the penetration of extraction solvents into the internal structure and therefore can enhance mass transfer, resulting in the increase of extraction yield^26^. Petcharat et al.^9^ found that increased yield of protein extracted from frog skin could be obtained with UAP at higher amplitude and longer ultrasonication time. In addition, Choi, et al.^27^ reported that UAP had an impact on alleviating protein extraction yield from Silkworm pupae where the highest yield was obtained with a 5 min ultrasonication time. Nevertheless, increasing of ultrasonication time to longer than 5 min did not significantly (*p* > 0.05) increase the extraction yield^27^. Our results confirm that UAP significantly (*p* ≤ 0.05) improved protein recovery of BL (12.03–15.57%), compared with that of TP (11.10%) regardless of the UAP condition used (Table 1). However, at the same amplitude, increased ultrasonication time did not effectively increase protein recovery, especially at longer than 20 min (*p* > 0.05). Notably, increases in amplitude had different impacts on protein recovery of BL depending on the ultrasonication time used and tended toward increased protein recovery when 30-min of ultrasonication time was used. On the contrary, with a 10-min ultrasonication time, there was a decrease in protein recovery with amplitude increased. It had been previously shown that the higher energy from ultrasound, particularly with higher amplitude and ultrasonication time, generally resulted in higher protein extraction yield from rice bran^10^, fish skin^9^ and edible insects^27^. However, Petcharat et al.^9^ showed that energy from UAP could destroy the insect powder matrix, in which some other co-extraction soluble solids could also be leached. This caused the dilution effect of extracted protein with the lower protein recovery. UAP could therefore be considered as an alternative technique to improve protein extraction from Bombay locust powder, where the UAP condition used directly governed the yield of solids and protein recovery.

**Table 1:** Solid yield and protein recovery of protein extracted from Bombay locust by ultrasound-assisted process under different conditions.

Samples		Solid yield (%)	Protein recovery (%)
TP		22.27 ± 0.30^f^	11.10 ± 1.06^d^
**40**	10	33.35 ± 0.24^d,z^	14.62 ± 0.06^ab,x^
	20	32.86 ± 0.74^de,y^	13.48 ± 0.35^bc,x^
	30	32.48 ± 0.15^e,z^	13.30 ± 0.16^bc,y^
**50**	10	34.39 ± 0.70^c,y^	14.23 ± 0.63^ab,x^
	20	35.36 ± 0.23^b,x^	14.48 ± 0.19^ab,x^
	30	34.69 ± 0.22^bc,y^	14.73 ± 1.24^ab,xy^
**60**	10	38.46 ± 0.23^a,x^	12.03 ± 0.94^cd^^,y^
	20	38.62 ± 0.38^a,x^	14.87 ± 1.22^ab,x^
	30	38.23 ± 0.38^a,x^	15.57 ± 0.92^a,x^

Data are expressed as mean ± standard deviation (n = 3).

TP, Typical extraction process (without ultrasound-assisted process).

40, 50, 60; Amplitudes of ultrasound-assisted process (%).

10, 20, 30; Ultrasonication times (min).

^a,b,c^^…^indicated significant differences of data in the same column (*P* < 0.05).

^x,y,z^ indicated significant differences of data comparing in the same ultrasonication time (*P* < 0.05).

### Surface hydrophobicity (SHP)

SHP is an index of the number of hydrophobic groups present on the surface of a protein molecule, which governs the protein conformation and its functional properties^8,28–30^. SHP of BL extracted by TP was 50.11, whereas those extracted by UAP showed significantly (*p* ≤ 0.05) higher SHP (61.52–74.16), regardless of UAP condition used (Table 2). When the same amplitude was used, SHP was significantly (*p* ≤ 0.05) increased with increasing ultrasonication time, especially at 40% amplitude. In contrast, the UAP with amplitudes higher than 40% resulted in significantly (*p* ≤ 0.05) decreased SHP with increase of ultrasonication time. Singh, et al.^8^ found that ultrasound had the potential on changing SHP of squid ovary protein by cavitation effect. This phenomenon facilitates the unfolding of protein molecules in which hydrophobic domains became more exposed during treatment. Similar result was reported by Arredondo-Parada, et al.^31^ who found that SHP of protein concentrate from giant squid (*Dosidicus gigas*) mantle increased after ultrasound treatment. On the other hand, under the harsh condition of UAP used it could cause excessive unfolding and rendered polymerization or aggregation of protein molecules via covalent or hydrophobic/hydrophobic interaction, which resulted in lowered SHP^7,9,29^. These effects could result in UAP at different amplitudes and time impacting on protein conformation, particularly SHP, which might govern its functional property and bioactivity of BL.

**Table 2:** Surface hydrophobicity and free alpha-amino group content of protein extracted from Bombay locust by ultrasound-assisted process under different conditions.

Samples		Surface hydrophobicity (S_0_ANS)	Free α-amino group content (mol/g solid)
TP		50.11 ± 1.00^g^	587.00 ± 0.16^d^
**40**	10	65.51 ± 0.19^e,z^	603.20 ± 0.09^b,x^
	20	67.48 ± 0.27^d,y^	599.40 ± 0.17^bc,x^
	30	69.41 ± 0.46^b,x^	597.60 ± 0.20^bc,y^
**50**	10	74.16 ± 0.62^a,x^	593.60 ± 0.16^c,y^
	20	68.81 ± 0.18^bc,x^	597.40 ± 0.21^bc,x^
	30	62.32 ± 0.44^f^^,y^	602.20 ± 0.09^b,y^
**60**	10	68.16 ± 0.41^cd^^,y^	598.00 ± 0.29^bc,x^
	20	64.68 ± 0.22^e,z^	602.20 ± 0.27^b,x^
	30	61.52 ± 0.99^f^^,y^	624.20 ± 0.17^a,x^

Data are expressed as mean ± standard deviation (n = 3).

TP, Typical extraction process (without ultrasound-assisted process).

40, 50, 60; Amplitudes of ultrasound-assisted process (%).

10, 20, 30; Ultrasonication times (min).

^a,b,c^^…^indicated significant differences of data in the same column (*P* < 0.05).

^x,y,z^indicated significant differences of data comparing in the same ultrasonication time (*P* < 0.05).

### Free alpha-amino group content

The BL from UAP at every condition tested showed significantly (*p* ≤ 0.05) higher α-amino group content than TP (Table 2). Comparing the same amplitude, free α-amino group content tended to increase with increase of ultrasonication time (*p* > 0.05), when UAP with an amplitude higher than 40% was conducted. In addition, the change of free α-amino group content could be varied, comparing with the same ultrasonication time, when the amplitude increased. At 20-min ultrasonication time, increase of amplitude could not affect free α-amino group content of PB, while 30-min ultrasonication time trended to increase free α-amino group content as increase of amplitude, particularly at 30 min (*p* ≤ 0.05). On the other hand, decreasing of free α-amino group content was observed when 10-min ultrasonication time was implied at the highest amplitude tested (*p* ≤ 0.05). The highest α-amino group content was observed from BL extracted by UAP with the highest severity tested (60% amplitude with 30 min ultrasonication time). These results from the present study were in accordance with those of Jambrak, et al.^32^, who indicated that the protein hydrolysis or degradation that occurred was due to the hydrodynamic forces of high pressure generated during microbubble collapse from the cavitation effect. As a consequence, the higher intensity of energy from ultrasound could render more cleavage and shortened peptides released^7,9^. However, the aggregation of liberated proteins could also form more stable particles, which would have more resistance to cavitation force and, consequently, cause lower protein degradation with lower α-amino group content of extracted protein^33^. It is also suggested that UAP could improve protein extraction efficacy from BL in which different conditions of UAP, particularly amplitudes and ultrasonication times, determined the protein degradation of resulting BL.

### Protein solubility

**Figure 1:**
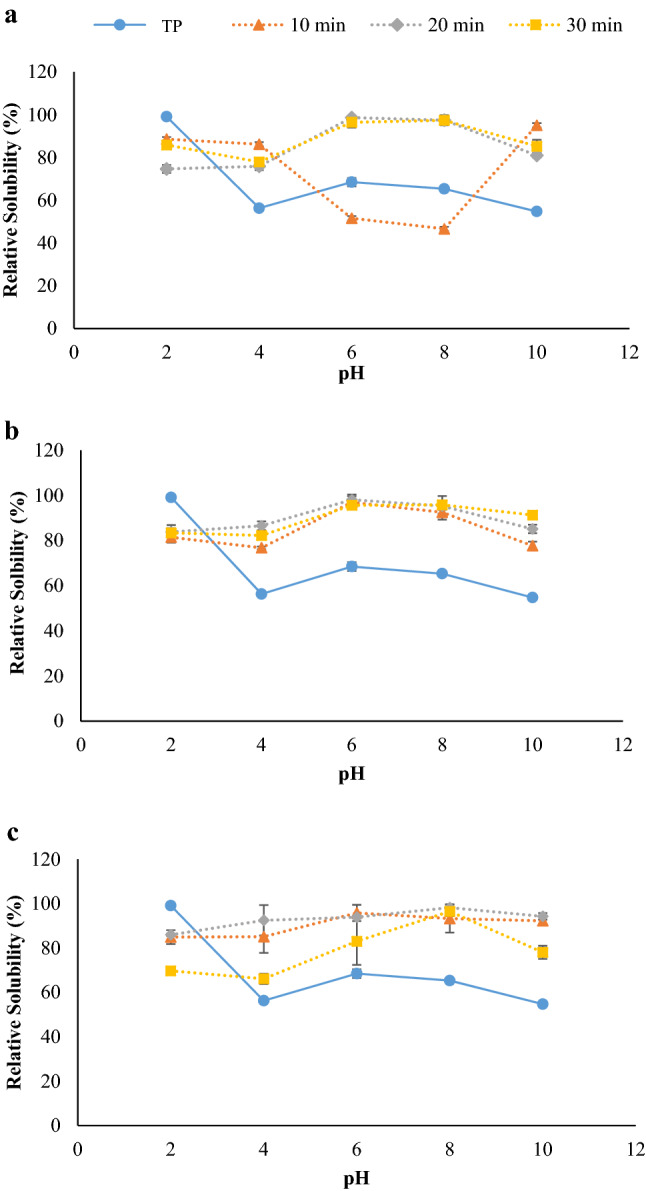
Relative solubility of protein extracted from Bombay locust by ultrasound-assisted process using 40% (**a**), 50% (**b**) and 60% (**c**) amplitudes at different ultrasonication times (10, 20, 30 min) in comparison with those from typical extraction process (TP). Bars represent the standard deviation (n = 3). Data obtained were analyzed by using One-way ANOVA.

There were significant differences (*p* ≤ 0.05) in protein solubility extracted by UAP at 40% (Fig. 1a), 50% (Fig. 1b) and 60% (Fig. 1c). The BL from TP had the highest solubility at pH 2 (99% relative solubility) and lowest solubility at the pH 4 (56% relative solubility). This result could be explained by the pH being close to the isoelectric point of protein, which would promote protein precipitation. The result was in accordance with findings of Chatsuwan, et al.^17^ who also reported the protein solubility of BL was lower as pH reached to 4. With UAP, protein solubility of BL was higher than those of TP, especially at pH levels higher than 2, regardless of UAP condition used. BL from UAP had protein solubility higher than 70% under the tested pH range. Nevertheless, protein solubility of BL was decreased when UAP at 40% amplitude for 10 min was conducted, compared with those from TP, particularly at pH 6–8 (*p* ≤ 0.05). The similar result was also observed for UAP with the highest severity (60% amplitude for 30 min), which showed significantly (*p* ≤ 0.05) lower solubility at pH in the range of 2–6, compared with others from the same amplitude tested. However, this BL still showed significantly (*p* ≤ 0.05) higher solubility than those obtained from TP, where the pH was higher than 2. This result was in accordance with previous studies that showed the impact of ultrasound on increasing the solubility of duck liver^29^, black bean^34^ and whey^32^. On the other hand, Singh, et al.^8^ reported that increases of amplitude and time of ultrasound treatment could also cause decreasing of protein solubility. This effect could be related to the hydrophobic surfaces of peptide chains by protein unfolding, resulting in protein aggregation and lowering the protein solubility^29^. Our results were in accordance with the change of surface hydrophobicity and free α-amino group content (Table 2) of BL from UAP, in which UAP governed protein conformation changes as well as low molecular weight peptide release. These effects could determine protein solubility and many functional applications of the extract, including emulsions, foams, gels and bioactivity of protein^7,17,35^. Therefore, UAP had a potential for enhancement of the protein solubility of BL, when the most appropriate conditions could be optimized and applied.

### Foaming properties

Foaming capacity (FC) and foam stability (FS) of BL extracted by TP were 114.00% and 100.56%, respectively. UAP significantly (*p* ≤ 0.05) improved FC (140.00–150.00%), but there were no significant effects between the UAP conditions tested (Table 3). However, FC tended to decrease with 30-min ultrasonication time when amplitude was increased. Similar results were also found for FS. UAP could effectively enhance FS of BL, compared with that of TP. Nevertheless, FS was not significantly (*p* ≤ 0.05) raised with UAP extraction under 40% amplitude for 10 min. It was found that 60% amplitude resulted in significant (*p* ≤ 0.05) decrease of FS, especially at the longest treatment time used, comparing with others at the same amplitude. Normally, proteins are more likely to become partially unfolded and hydrolyzed to small peptides via the cavitation effect. This could affect protein molecule rearrangement at the interface between aqueous and air phases and form a stable film surrounding the air bubble in foam system^32,36^. However, the harsh condition of ultrasound could cause excessive unfolding of protein resulting in the loss of solubility and increased drainage of water from lamella film, resulting in decrease of FS^8^. Similar results were reported on the FS of squid ovary powder^37^ and protein from album seeds (*Chenopodium album*)^38^. UAP could cause the decrease of foaming property depending on the conditions of ultrasound treatment used. Therefore, the application of UAP for extraction of BL, under the appropriate conditions should ensure that a good recovery is achieved with appropriate functional properties, particularly foaming capacity and foaming stability.

**Table 3:** Foaming and emulsifying properties of protein extracted from Bombay locust by ultrasound-assisted process under different conditions.

Samples		FC (%)	FS (%)	EAI (m^2^/g)	ESI (min)
TP		114.00 ± 3.85^b^	100.56 ± 0.00^d^	8.46 ± 0.44^bc^	18.35 ± 2.83^b^
**40**	10	140.00 ± 8.82^a,x^	103.33 ± 0.00^d,z^	9.39 ± 0.60^ab,x^	16.44 ± 1.25^bc,x^
	20	145.56 ± 5.09^a,x^	116.67 ± 0.00^a,x^	9.62 ± 0.42^a,x^	20.62 ± 2.61^a,x^
	30	150.00 ± 0.00^a,x^	116.67 ± 0.00^a,x^	8.89 ± 1.24^abc,x^	16.35 ± 1.51^bc,x^
**50**	10	150.00 ± 0.00^a,x^	116.67 ± 0.00^a,x^	8.37 ± 0.83^c,x^	15.42 ± 0.30^c,x^
	20	148.89 ± 1.92^a,x^	116.67 ± 0.00^a,x^	8.23 ± 0.32^c,y^	16.16 ± 0.68^bc,y^
	30	145.56 ± 5.09^a,xy^	116.67 ± 0.00^a,x^	9.72 ± 0.23^a,x^	16.23 ± 1.02^bc,x^
**60**	10	140.00 ± 0.00^a,x^	106.67 ± 0.00^c,y^	9.00 ± 0.35^abc,x^	15.36 ± 0.68^c,x^
	20	140.00 ± 17.32^a,x^	113.33 ± 5.77^a,x^	9.48 ± 0.25^a,x^	15.37 ± 0.53^c,y^
	30	142.22 ± 3.85^a,y^	110.00 ± 0.00^b,y^	9.17 ± 0.48^abc,x^	16.31 ± 0.95^bc,x^

Data are expressed as mean ± standard deviation (n = 3).

FC, foaming capacity; FS, foam stability; EAI, emulsion activity index; ESI, emulsion stability index; TP, Typical extraction process (without ultrasound-assisted process).

40, 50, 60; Amplitudes of ultrasound-assisted process (%).

10, 20, 30; Ultrasonication times (min).

^a,b,c^^…^indicated significant differences of data in the same column (*P* < 0.05).

^x,y,z^indicated significant differences of data comparing in the same ultrasonication time (*P* < 0.05).

### Emulsifying properties

Emulsion activity index (EAI) and emulsion stability index (ESI) are used to indicate the emulsifying properties of protein by using the oil droplet distribution and absorption ability of protein at its interface^39,40^. Our results showed that UAP had no adverse effect on EAI, regardless of the conditions tested. They were all in the range of 8.23–9.72 m^2^/g, while that of TP was 8.46 m^2^/g (Table 3). It was noted that UAP at every amplitude tested showed improved EAI depending on ultrasonication time applied. UAP at 40% and 60% amplitude for 20 min showed significant increases (*p* ≤ 0.05) in EAI, compared with that of TP, while a longer ultrasonication time was required for 50% amplitude. On the other hand, some of the conditions used for UAP in the present study showed the detrimental effect on ESI compared with that of TP, especially when the amplitude was higher than 40%. Nevertheless, ESI of BL from those amplitudes, under suitable ultrasonication times, were comparable to that of TP (*p* > 0.05). For example, UAP at 50% amplitude should be applied for longer than 10 min, while 60% amplitude should be applied at the longest period tested (30 min). These results suggest that increases in EAI and ESI could be related to increase of surface hydrophobicity of BL extracted with UAP (Table 2). The degree of protein unfolding (partial/fully) of protein induced by UAP was likely responsible for the adsorption at the oil droplets interface. At the optimal unfolding, the exposed reactive groups (e.g. hydrophobic and sulfhydryl residues) may have helped to stabilise the protein network at the interface, and thus helping the stabilisation of the emulsion. Protein unfolding determined hydrophobic and hydrophilic surface ratio of protein molecules, which was associated with their ability to rearrange and form a stable layer at their interface^8,28,41^. In addition, shear force and shock waves generated by UAP can cause protein cleavage and liberate shortened peptides, which increases free α-amino group content (Table 1). This would improve their surface adsorption properties^25,42,43^. The excessive cleavage could also lower the capability of emulsifier protein to reduce interfacial tension and stabilizing emulsion systems. It was reported that the formation of low molecular weight peptides could exceed aggregation of unfolded protein that exist in the harsh conditions of UAP. This effect could reduce the ability of protein reorientation at interface, resulting a decrease in the emulsion property of proteins^5,44,45^. Moreover, UAP could led the changes in chemical reactions, particularly protein and lipid oxidation resulting the oxidation products such as cross-links formed compounds, which determined the functional properties of the obtained protein^8,23^. Therefore, the conditions of UAP used during protein extraction played an important role in emulsion properties of the resulting proteins. Under appropriate amplitude and ultrasonication time, UAP was shown to improve the surface activity of BL, particularly their emulsion properties, which could be used as a functional ingredient in emulsion foods.

### Antioxidative activities

**Figure 2:**
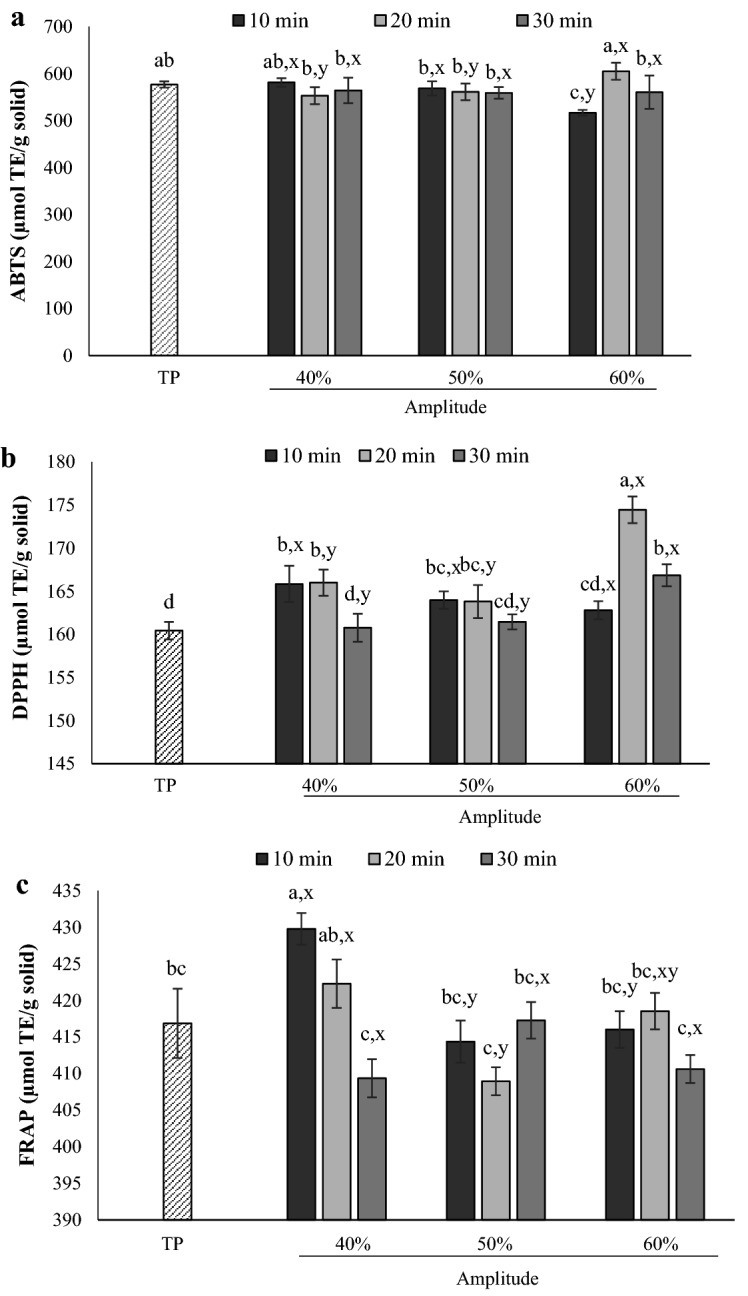
Antioxidant activity of protein extracted from Bombay locust by ultrasound-assisted process under different amplitudes (40, 50, 60%) and ultrasonication times (10, 20, 30 min) in comparison with those from typical extraction process (TP). ABTS radical scavenging activity (ABTS) (**a**), DPPH radical scavenging activity (DPPH) (**b**), Ferric reducing antioxidant power (FRAP) (**c**). Bars represent the standard deviation (n = 3). ^a,b,c^indicated significant differences in values between all data (*p* ≤ 0.05). ^x,y,z^ indicated significant differences of data comparing in the same ultrasonication time (*p* ≤ 0.05). Data obtained were analyzed by using One-way ANOVA.

For ABTS radical scavenging activity, there was no significant differences (*p* > 0.05) of BL from either TP or UAP for all conditions tested (Fig. 2a). However, UAP with 60% amplitude for 10 min (UAP-60/10) had detrimental effects on ABTS radical scavenging activity of BL, which was significantly (*p* ≤ 0.05) lower than that of TP. When compared in the same amplitude, increasing ultrasonication time had no adverse effect on ABTS activity of BL, especially at 40% and 50% amplitudes. At the same ultrasonication time, the change of ABTS activity was varied depending on the amplitude used. Increase of ABTS activity was observed when 20-min ultrasonication was conducted, especially at 60% amplitude, while 10-min ultrasonication tended to decrease of ABTS activity with increase of amplitude. On the other hand, increase of amplitude had no impact on ABTS activity of BL when 30-min ultrasonication time was applied (*p* > 0.05). UAP showed some improvement on DPPH radical scavenging of BL depending on the conditions used, compared with that of TP (Fig. 2b). Moreover, UAP resulted in higher DPPH activity than TP (160.44 µmol TE/g solid), especially that from UAP-60/20, which had the highest DPPH activity (174.44 µmol TE/g solid). Similar result was observed for FRAP. Under various conditions of UAP, the resulting BL had significantly (*p* > 0.05) higher FRAP activity than TP (Fig. 2c), particularly at 40% amplitude for 10 min (UAP-40/10). However, a significant decrease (*p* ≤ 0.05) in FRAP activity was detected for UAP at 40% amplitude with increased ultrasonication time. On the other hand, increased ultrasonication time had no significant (*p* > 0.05) impact on FRAP from UAP with 50% and 60% amplitude. At the same treatment time, increasing amplitude used tended to decrease FRAP activity, particularly with 40–50% amplitude. Klompong et al.^46^ commented that the configurations of protein, such as shape, size, and surface hydrophobicity, could govern the antioxidative activity, in which changes in protein configurations directly affect antioxidative function of peptides. UAP caused protein unfolding, consequently exposing hydrophobic regions, in which low molecular weight peptides could also be generated in some degree, depending on the severity of UAP condition used^7,8,13^. Our results support this effect on surface hydrophobicity and free α-amino group content (Table 2). Gülseren et al.^47^ and Chen, et al.^48^ also reported that the expression of peptide chain linked in the hydrophobic region of proteins affects its antioxidant activity. Liu et al.^49^ working with silkworm moth (*Bombyx mori*) reported that lower molecular weight peptides had higher antioxidative activity, and showed more amino acids exposure that could interact with free radicals. Mintah et al.^13^ working with black soldier fly larvae (*Hermetia illucens*) found that the difference in ultrasound condition used determined the variations of antioxidative activity of the extracted protein. It was also reported that those variations were governed by amino acid compositions of the relative peptides^50^. These results suggested that the UAP process used strongly determined the antioxidative activities of the BL protein extract.

Overall, UAP could be effectively applied to extract protein from BL giving a high extraction yield. Also, the conditions of UAP used showed marked impacts on both functional and antioxidative properties of the extract. UAP at 60% amplitude for 20 min augmented the extraction efficiency giving protein with solubility, foaming properties, emulsifying properties and antioxidative activity comparable to or better than those from TP. Therefore, BL prepared with this optimum condition was selected and used in the subsequent studies on its molecular characteristics in comparison with that of protein extracted by TP.

### Zeta potential

**Figure 3:**
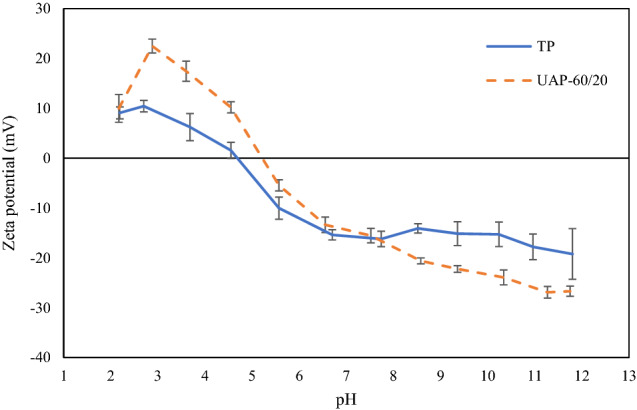
Zeta (ζ)-potential of protein extracted from Bombay locust by selected ultrasound-assisted process at 60% amplitude for 20 min (UAP-60/20) and typical extraction process (TP) at different pH values. Bars represent the standard deviation (n = 3).

Substances with high zeta potential have highly charged particles that can prevent aggregation due to electric repulsion. Low zeta potential results in attraction being higher than repulsion which can result in coagulation. BL from UAP-60/20 had a different zeta potential profile, compared with that from TP (Fig. 3). Moreover, the isoelectric point of BL from TP was 4.76 and it increased to 5.27 for UAP-60/20. This indicated the increase of surface net charge due to the UAP used, especially at pH levels lower than 6 or higher than 8, compared with those from TP. This could favor dissolution/dissolution-related functional characteristics of proteins^6^. Similar results were also reported for milk and pea protein^44^, solder fly larvae^6^, frog skin gelatin^7^ and fish skin collagen^9^. Protein configurations were modified by ultrasound treatment, which promoted the exposure of peptides molecules on the protein surface, zeta-potential and their functional properties. Therefore, the application of UAP for BL extraction affected the change of the zeta potential profile and isoelectric point of resulting protein, which determined its characteristics and applicability to food processing systems.

### Fourier transform infrared (FTIR) spectroscopy

**Figure 4:**
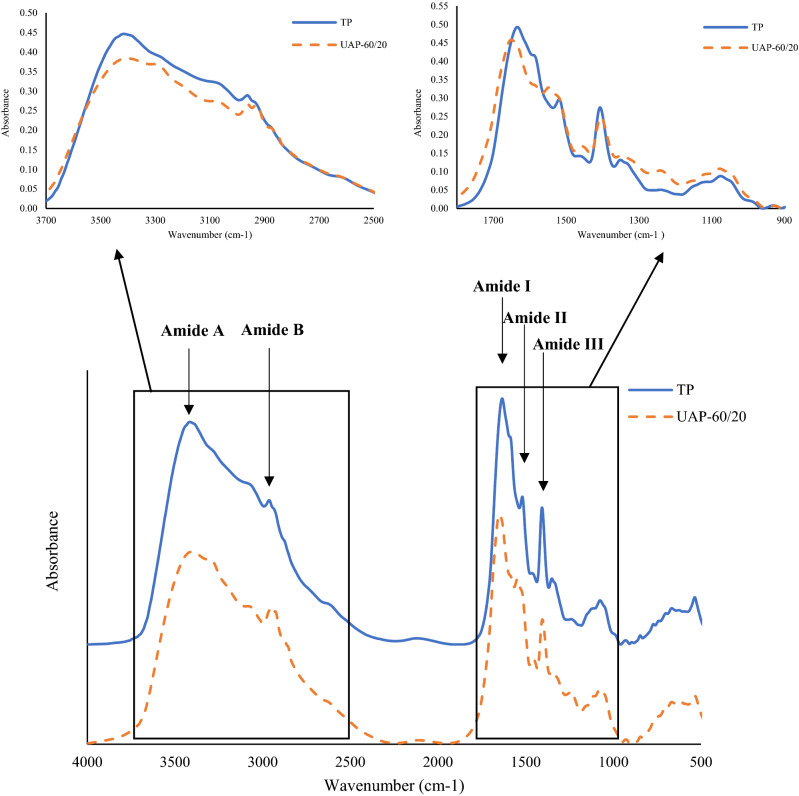
FTIR spectra of protein extracted from Bombay locust by selected ultrasound-assisted process at 60% amplitude for 20 min (UAP-60/20) and typical extraction process (TP).

FTIR spectrums of BL protein extracted by UAP with 60% amplitude for 20 min (UAP-60/20) and by TP were both in accordance to those found from other edible insects (Fig. 4)^6,51–53^. They both showed the dominant band for Amide A (3300–3440 cm^−1^) and B (2900–3200 cm^−1^). Also, Amide I (1700–1600 cm^−1^), II (1600–1500 cm^−1^) and III (1300–1200 cm^−1^) bands were observed with minor band at 1200–1000 cm^−1^, which were attributed to carbohydrates^54^. Amide A and B correspond to stretching vibrations of N–H and C–H as well as –NH_3_, respectively^55^. Amide I represented C=O stretching vibrations coupled to N–H bending vibrations, CN stretch and CCN deformation^56^. Bending vibration of N–H coupled to C–N is represented by Amide II band. Amide III band indicates a complex mix of α-helix and β-sheet along with random coil of protein structure^56^. It was noted that Amide A and B bands of BL obtained from TP and UAP-60/20 were both at a similar wave number (3404 and 2955 cm^−1^, respectively). Nevertheless, the lower amplitude of the Amide A and B regions was observed for BL from UAP-60/20. It might be postulated that protein–protein interactions via a hydrogen bond occurred to a higher degree during ultrasound treatment, leading to lower peak in intensity of Amide A and B. Moreover, lowered Amide I band intensity with the higher wave number (1649 cm^−1^) was found for BL with UAP-60/20, compared with those from TP, which was 1628 cm^−1^. These results suggested that there was less expression of C=O group, indicating the protein molecule was more compact, where inter-molecular cross-link via protein aggregation might have occurred because of the ultrasound treatment^7^. Furthermore, the generation of protein fragments, as influenced by UAP, could also shift an Amide I band to a higher wave number^9,57^. Amide II of BL from UAP-60/20 showed higher amplitude with a higher wave number (1541 cm^−1^), which suggested greater loss of H-bonding between adjacent chains via NH groups^9^. Amide III was observed at 1400 cm^−1^ for samples of both extraction methods, with the lower amplitude found from UAP-60/20. This indicated a higher proportion of disordered structure from α-helical to random coils with a lower degree of ordered structure in the BL extract after being exposed to UAP-60/20^57^. Thus, the selected UAP (60% amplitude for 20 min) had a clear impact on the molecular structure and functional groups of BL extract, which could provide high extraction yield with good functional and antioxidative properties.

### Amino acid composition

BL extracted protein contained 10 essential amino acids and nine non-essential amino acids. The most abundant of the essential amino acids were arginine, leucine, and histidine, respectively. Glutamine/glutamic acid, aspartic acid/asparagine and alanine were major non-essential amino acid composition (Table 4). This result is in accordance with the previous works by Chatsuwan et al.^17^ and Köhler et al.^2^ who also reported the amino acids composition in Bombay locust protein. BL had significant amounts of hydrophobic residues such as leucine, alanine, proline, valine, phenylalanine, isoleucine, threonine and methionine and the basic amino acids including histidine and lysine, which had previously been reported for their antioxidative activity^2,49,58^. The presence of these amino acids in peptide sequences has been commonly linked to the antioxidative properties of peptides^59^. Among non-essential amino acids, glutamic acid, aspartic acid, alanine, and glycine are classified as delicious amino acid (DAA) which play a significant role in the umami flavour^60^. From the results, the DAA content of a BL protein obtained from UAP-60/20 was 18.27% of the total amino acid content. Thus, the protein from BL could have the characteristic umami flavour. Overall, BL obtained from UAP-60/20 is a potential source of nutrients, particularly amino acids, for human consumption since they have good techno-functional and antioxidative properties.

**Table 4:** Amino acid composition of protein extracted from Bombay by selected ultrasound-assisted process at 60% amplitude for 20 min.

Amino acids	Content (g/100 g)
**Essential amino acid**	
Arginine	10.9 ± 0.00
Histidine	4.28 ± 0.00
Isoleucine	2.32 ± 0.00
Leucine	5.41 ± 0.00
Lysine	3.04 ± 0.00
Methionine	1.4 ± 0.00
Phenylalanine	2.42 ± 0.00
Threonine	2.29 ± 0.00
Tryptophan	0.71 ± 0.00
Valine	2.67 ± 0.00
*Total essential amino acid*	35.44 ± 0.00
**Non-essential amino acid**	
Alanine	4.41 ± 0.00
Aspartic acid/asparagine	5.38 ± 0.00
Glutamine/glutamic acid	7.53 ± 0.00
Glycine	0.95 ± 0.00
Proline	2.88 ± 0.00
Serine	2.49 ± 0.00
Tyrosine	0.78 ± 0.00
Cysteine	1.15 ± 0.00
Hydroxylysine	ND
Hydroxyproline	0.12 ± 0.00
*Total non-essential amino acid*	25.69 ± 0.00

ND, not detected.

## Conclusion

UAP under appropriated condition could effectively improve the protein extraction from Bombay locust powder, compared to a traditional extraction method. The different amplitudes and ultrasonication times had a crucial impact on the extraction yield, functional and antioxidative activity. The appropriate UAP condition should be concerned to minimize detrimental effects on the molecular structure of the protein. UAP at 60% amplitude for 20 min could be a promising condition for extracting the protein from Bombay locust with less adverse effect. Therefore, the ultrasound-assisted process showed good potential for being used in the food processing industry for protein extraction from Bombay locust, and possibly other edible insects, which could provide a method of enhancing protein content that could be used in functional foods.

## References

[1] Zielińska E, Karaś M, Baraniak B (2018). Comparison of functional properties of edible insects and protein preparations thereof. LWT..

[2] Köhler R, Kariuki L, Lambert C, Biesalski HK (2019). Protein, amino acid and mineral composition of some edible insects from Thailand. J. Asia Pac. Entomol..

[3] Paul A (2017). Insect fatty acids: A comparison of lipids from three Orthopterans and *Tenebrio molitor* L. larvae. J. Asia Pac. Entomol..

[4] Yi L (2013). Extraction and characterisation of protein fractions from five insect species. Food Chem..

[5] Zhao X, Vázquez-Gutiérrez JL, Johansson DP, Landberg R, Langton M (2016). Yellow mealworm protein for food purposes-extraction and functional properties. PLoS ONE.

[6] Mintah BK (2020). Characterization of edible soldier fly protein and hydrolysate altered by multiple-frequency ultrasound: Structural, physical, and functional attributes. Process Biochem..

[7] Karnjanapratum S, Benjakul S (2020). Asian bullfrog (*Rana tigerina*) skin gelatin extracted by ultrasound-assisted process: Characteristics and *in-vitro* cytotoxicity. Int. J. Biol. Macromol..

[8] Singh A, Benjakul S, Kijroongrojana K (2018). Effect of ultrasonication on physicochemical and foaming properties of squid ovary powder. Food Hydrocoll..

[9] Petcharat T, Benjakul S, Karnjanapratum S, Nalinanon S (2020). Ultrasound-assisted extraction of collagen from clown featherback (*Chitala ornata*) skin: Yield and molecular characteristics. J. Sci. Food Agric..

[10] Ly H, Tran T, Tran T, Ton N, Le V (2018). Application of ultrasound to protein extraction from defatted rice bran. Int. Food Res. J..

[11] Mishyna M, Martinez J-JI, Chen J, Benjamin O (2019). Extraction, characterization and functional properties of soluble proteins from edible grasshopper (*Schistocerca gregaria*) and honey bee (*Apis mellifera*). Food Res. Int..

[12] Gulzar S, Benjakul S (2020). Characteristics and storage stability of nanoliposomes loaded with shrimp oil as affected by ultrasonication and microfluidization. Food Chem..

[13] Mintah BK (2019). Techno-functional attribute and antioxidative capacity of edible insect protein preparations and hydrolysates thereof: Effect of multiple mode sonochemical action. Ultrason. Sonochem..

[14] Kim S-Y, Kim M-J, Jung S-K, Kim H-Y (2019). Development of a fast real-time PCR assay based on TaqMan probe for identification of edible rice grasshopper (*Oxya chinensis*) in processed food products. Food Res. Int..

[15] Lombardi A, Vecchio R, Borrello M, Caracciolo F, Cembalo L (2019). Willingness to pay for insect-based food: The role of information and carrier. Food Qual. Prefer..

[16] Indriani S, Bin Ab Karim MS, Nalinanon S, Karnjanapratum S (2020). Quality characteristics of protein-enriched brown rice flour and cake affected by Bombay locust (*Patanga succincta* L.) powder fortification. LWT..

[17] Chatsuwan N, Nalinanon S, Puechkamut Y, Lamsal BP, Pinsirodom P (2018). Characteristics, functional properties, and antioxidant activities of water-soluble proteins extracted from grasshoppers, *Patanga succincta* and *Chondracris roseapbrunner*. J. Chem..

[18] Horwitz W, Latimer GW (2005). AOAC Official Methods of Analysis of AOAC International.

[19] Karnjanapratum S, Benjakul S (2015). Cryoprotective and antioxidative effects of gelatin hydrolysate from unicorn leatherjacket skin. Int. J. Refrig..

[20] Benjakul S, Morrissey MT (1997). Protein hydrolysates from Pacific whiting solid wastes. J. Agric. Food Chem..

[21] Lowry OH, Rosebrough NJ, Farr AL, Randall RJ (1951). Protein measurement with the Folin phenol reagent. J. Biol. Chem..

[22] Binsan W (2008). Antioxidative activity of Mungoong, an extract paste, from the cephalothorax of white shrimp (*Litopenaeus vannamei*). Food Chem..

[23] Karnjanapratum S, Sinthusamran S, Sae-leaw T, Benjakul S, Kishimura H (2017). Characteristics and gel properties of gelatin from skin of Asian Bullfrog (*Rana tigerina*). Food Biophys..

[24] Steel RG, Torrie JH (1986). Principles and Procedures of Statistics: A Biometrical Approach.

[25] Jain S, Anal AK (2016). Optimization of extraction of functional protein hydrolysates from chicken egg shell membrane (ESM) by ultrasonic assisted extraction (UAE) and enzymatic hydrolysis. LWT.

[26] Gogate PR, Pandit AB (2010). Theoretical and Experimental Sonochemistry Involving Inorganic Systems.

[27] Choi BD, Wong NA, Auh J-H (2017). Defatting and sonication enhances protein extraction from edible insects. Korean J. Food Sci. Anim. Resour..

[28] Quan TH, Benjakul S (2019). Duck egg albumen hydrolysate-epigallocatechin gallate conjugates: Antioxidant, emulsifying properties and their use in fish oil emulsion. Colloid. Surf. A Physicochem. Eng. Asp..

[29] Zou Y (2017). Effects of ultrasound assisted extraction on the physiochemical, structural and functional characteristics of duck liver protein isolate. Process Biochem..

[30] Zou Y (2019). Effect of different time of ultrasound treatment on physicochemical, thermal, and antioxidant properties of chicken plasma protein. Poult. Sci..

[31] Arredondo-Parada I (2020). Effect of ultrasound on physicochemical and foaming properties of a protein concentrate from giant squid (*Dosidicus gigas*) mantle. LWT..

[32] Jambrak AR, Mason TJ, Lelas V, Herceg Z, Herceg IL (2008). Effect of ultrasound treatment on solubility and foaming properties of whey protein suspensions. J. Food Eng..

[33] Lomakina K, Mikova K (2006). A study of the factors affecting the foaming properties of egg white—A review. Czech J. Food Sci..

[34] Jiang L (2014). Effects of ultrasound on the structure and physical properties of black bean protein isolates. Food Res. Int..

[35] Sripokar P, Benjakul S, Klomklao S (2019). Antioxidant and functional properties of protein hydrolysates obtained from starry triggerfish muscle using trypsin from albacore tuna liver. Biocatal. Agric. Biotechnol..

[36] Chen Y, Sheng L, Gouda M, Ma M (2019). Impact of ultrasound treatment on the foaming and physicochemical properties of egg white during cold storage. LWT..

[37] Singh A, Benjakul S, Karnjanapratum S (2019). Use of ultrasonicated squid ovary powder as a replacer of egg white powder in cake. J. Food Sci. Technol..

[38] Mir NA, Riar CS, Singh S (2019). Physicochemical, molecular and thermal properties of high-intensity ultrasound (HIUS) treated protein isolates from album (*Chenopodium album*) seed. Food Hydrocoll..

[39] Higuera-Barraza OA (2017). Effect of pulsed ultrasound on the physicochemical characteristics and emulsifying properties of squid (*Dosidicus gigas*) mantle proteins. Ultrason. Sonochem..

[40] Thiansilakul Y, Benjakul S, Shahidi F (2007). Compositions, functional properties and antioxidative activity of protein hydrolysates prepared from round scad (*Decapterus maruadsi*). Food Chem..

[41] Bing L, Haji Akber A, Abulimiti Y (2019). Optimization of ultrasound-assisted extraction of sheep abomasum protein concentrates by response surface methodology and evaluation of their properties. Food Sci. Technol..

[42] Liu L (2019). Effect of ultrasound assisted heating on structure and antioxidant activity of whey protein peptide grafted with galactose. LWT..

[43] Jambrak AR, Mason TJ, Lelas V, Paniwnyk L, Herceg Z (2014). Effect of ultrasound treatment on particle size and molecular weight of whey proteins. J. Food Eng..

[44] O'Sullivan J, Arellano M, Pichot R, Norton I (2014). The effect of ultrasound treatment on the structural, physical and emulsifying properties of dairy proteins. Food Hydrocoll..

[45] Hall FG, Jones OG, O'Haire ME, Liceaga AM (2017). Functional properties of tropical banded cricket (*Gryllodes sigillatus*) protein hydrolysates. Food Chem..

[46] Klompong V, Benjakul S, Kantachote D, Shahidi F (2007). Antioxidative activity and functional properties of protein hydrolysate of yellow stripe trevally (*Selaroides leptolepis*) as influenced by the degree of hydrolysis and enzyme type. Food Chem..

[47] Gülseren İ, Güzey D, Bruce BD, Weiss J (2007). Structural and functional changes in ultrasonicated bovine serum albumin solutions. Ultrason. Sonochem..

[48] Chen H-M, Muramoto K, Yamauchi F (1995). Structural analysis of antioxidative peptides from Soybean. beta.-Conglycinin. J. Agric. Food Chem..

[49] Liu Y, Wan S, Liu J, Zou Y, Liao S (2017). Antioxidant activity and stability study of peptides from enzymatically hydrolyzed male silkmoth. J. Food Process Preserv..

[50] Wu H-C, Chen H-M, Shiau C-Y (2003). Free amino acids and peptides as related to antioxidant properties in protein hydrolysates of mackerel (*Scomber austriasicus*). Food Res. Int..

[51] Chatsuwan N, Puechkamut Y, Pinsirodom P (2018). Characterization, functionality and antioxidant activity of water-soluble proteins extracted from *Bombyx mori* Linn. CAST.

[52] Horvatinec J, Svečnjak L (2020). Infrared (FTIR) spectral features of honey bee (*Apis mellifera* L.) hemolymph. J. Cent. Eur. Agric..

[53] Mellado-Carretero J (2020). Rapid discrimination and classification of edible insect powders using ATR-FTIR spectroscopy combined with multivariate analysis. J. Insects Food Feed..

[54] Talari ACS, Martinez MAG, Movasaghi Z, Rehman S, Rehman IU (2017). Advances in Fourier transform infrared (FTIR) spectroscopy of biological tissues. Appl. Spectrosc. Rev..

[55] Ramappa VK, Dev P, Shankar S, Shanker U (2016). Analysis of chemical compounds in different mulberry and non mulberry silkworm pupae powder by FTIR and EDX. Int. J. Sci. Innov. Res..

[56] Du M (2018). Extraction, physicochemical characteristics and functional properties of Mung bean protein. Food Hydrocoll..

[57] Kittiphattanabawon P, Benjakul S, Sinthusamran S, Kishimura H (2016). Gelatin from clown featherback skin: Extraction conditions. LWT.

[58] Wang W, De Mejia EG (2005). A new frontier in soy bioactive peptides that may prevent age-related chronic diseases. Compr. Rev. Food Sci. Food Saf..

[59] Megías C (2004). Purification of an ACE inhibitory peptide after hydrolysis of sunflower (*Helianthus annuus* L.) protein isolates. J. Agric. Food Chem..

[60] Machado M (2020). Amino acid profile and protein quality assessment of macroalgae produced in an integrated multi-trophic aquaculture system. Foods.

